# Alteration of DNA mismatch repair capacity underlying the co-occurrence of non-small-cell lung cancer and nonmedullary thyroid cancer

**DOI:** 10.1038/s41598-021-83177-1

**Published:** 2021-02-11

**Authors:** Shiro Fujita, Katsuhiro Masago

**Affiliations:** 1 Department of Respiratory Medicine, Kobe Central Hospital, 2-1-1 Soyama-cho, Kita-ku, Kobe, Hyogo 651-1145 Japan; 2 Division of Integrated Oncology, Institute of Biomedical Research and Innovation, 2-2 Minatojima Minami-machi, Chuo-ku, Kobe, Hyogo 650-0047 Japan; 3 Department of Pathology and Molecular Diagnostics, Aichi Cancer Center, 1-1 Kanokoden, Chikusa-ku, Nagoya, Aichi 464-8681 Japan

**Keywords:** Cancer, Genetics, Diseases, Oncology

## Abstract

Both non-small-cell lung cancer cases in never-smokers and nonmedullary thyroid cancer cases have been increasing in developed countries. Some studies have shown an excess of co-occurrence of non-small-cell lung cancer and nonmedullary thyroid cancer. We aimed to clarify the underlying genetic factors that contribute to the occurrence of these two malignancies. We performed germline exome sequencing in a cohort of 9 patients with the two malignancies. In terms of candidate genes, we performed target resequencing, immunohistochemistry, and microsatellite instability testing on another cohort. Two rare missense heterozygous variants in *MSH6* were identified and verified by Sanger sequencing. One available tumour specimen showed heterogeneous MSH6 status in immunohistochemistry. Further exploration with different cohorts (a total of 8 patients with the two malignancies) demonstrated that 2 out of 8 patients had a germline missense or promotor variant of *MLH1* and four out of 10 tumour specimens revealed heterogeneous immunohistochemistry staining in any of the four mismatch repair proteins: MLH1, PMS2, MSH2 and MSH6. Although our cohort showed a different disease profile than Lynch syndrome, this study suggests causal roles of impaired DNA mismatch repair capacity in non-small-cell lung cancer and nonmedullary thyroid cancer.

## Introduction

Lung cancer, which occurred in approximately 2.1 million patients and caused an estimated 1.8 million deaths worldwide in 2018, constitutes a substantial disease burden^1,2^. Lung cancer is a malignant tumour derived from intrathoracic respiratory organs such as bronchi and lung parenchyma but is not a uniform disease. In terms of the histologic classification of lung cancer, the relative incidence of adenocarcinoma has risen in the past 20 years and there has been a corresponding decrease in the incidence of other types of non-small-cell lung cancer (NSCLC) and small cell lung cancer^3^. The reasons for the relative increase in lung adenocarcinoma are not yet clear. One explanation is the introduction of low-tar filter cigarettes in the 1960s, and another is that lung adenocarcinoma in never-smokers has been increasing^4^. Although tobacco smoking is the major risk factor accounting for most lung cancer diagnoses, a growing body of evidence suggests that a substantial number of patients (15% to 20% of cases in men and over 50% in women) who suffer from NSCLC are never-smokers^4–6^. In addition to the aforementioned predominant adenocarcinoma histology, lung adenocarcinoma in patients without a history of smoking has a higher frequency of positive cases of particular driver gene alterations (e.g., EGFR gene mutation and ALK gene rearrangement) than lung adenocarcinoma in ever-smokers. These biologically distinct characteristics cause clinicians to take a different approach in treating these cancers, especially in cases of advanced-stage disease, where systemic chemotherapy (including molecular targeted therapy and immune checkpoint blockade therapy) is the mainstay treatment^7,8^.

Lung cancer, which was once thought to be solely attributable to environmental exposure, has been studied for its associated genetic factors. On the basis of a pooled analysis from the International Lung Cancer Consortium, individuals with a first-degree relative who suffered from lung cancer had a 1.51-fold increase in the risk of lung cancer, after adjustment for smoking and other potential confounders (95% confidence interval (CI) 1.39–1.63)^9^. In another meta-analysis, lung cancer risks were stratified according to the number of affected relatives. The pooled relative risk of lung cancer associated with a single affected relative was 1.57 (95% CI 1.34–1.84), and for two or more affected relatives, it was 2.52 (95% CI 1.72–3.70)^10^. Genome-wide association studies have also identified genetic factors associated with various human diseases, including lung cancer. The loci of more than 40 genes have been associated with lung cancer risk since the first genome-wide association study results for lung cancer were reported in 2008^11–13^. However, many of these candidate genes are still under investigation, and only a few germline mutations have been shown to confer an inherent predisposition to lung cancer.

Thyroid cancer is the most common malignancy of the endocrine system, and its incidence has increased rapidly in recent decades worldwide. In Japan, the age-adjusted thyroid cancer incidence rate increased: from 2.1 (men) and 9.3 (women) per 100,000 person-years in 1990 to 4.1 and 12.3 per 100,000 person-years, respectively, in 2012^14^. The vast majority of thyroid cancer is of nonmedullary histology. Although most nonmedullary thyroid cancers (NMTC) occur in a sporadic fashion, approximately 5% to 10% of NMTC patients have a family history of NMTC, and a history of NMTC in a first-degree relative is reported to increase the risk. In one study, there was an approximately tenfold increased risk of NMTC in relatives of NMTC patients^15^. A second study found that the standardized incidence ratio for NMTC was 3 to 11 in family members with a first-degree involved relative^16^. However, the aetiology of these cancers is largely unknown, except for rare familial tumour syndrome (e.g., familial adenomatous polyposis, Gardner’s syndrome, Cowden’s disease).

The development of multiple cancers in the same patient has been widely documented and can result from various causes, including inherited predisposition. Despite the increased risk of upper aerodigestive tract cancers and urinary tract cancers due to the harmful effects of smoking, some studies have shown excess co-occurrence of lung and thyroid cancers^17–21^. A recent study of coexisting NSCLC (including lung adenocarcinoma) and NMTC in the same never-smoking patients supports a genetic predisposition underlying the association between these two cancers^21^.

To clarify the underlying genetic factors that contribute to the occurrence of these malignancies, we conducted exome sequencing of DNA extracted from leukocytes of 9 patients with NMTC and NSCLC. Two patients harboured *MSH6* missense mutations, and immunohistochemistry (IHC) of the tumour tissue of one patient revealed abnormal staining because of heterogeneous patterns of MSH6 loss, which implies differences in mismatch repair (MMR) status. In addition, we found 8 cases for which tissue specimens were available and found similar findings suggesting changes in MMR.

## Patients and methods

### Patients

The study was conducted in accordance with the ethical principles of the Declaration of Helsinki, following the signature of written informed consent by the patients and the approval by the Research Ethics Committee of the Institute of Biomedical Research and Innovation. All patients were enrolled in our study between January 2013 and June 2017. Both NSCLC and NMTC were confirmed histologically, regardless of whether they were synchronous or metachronous. The first cohort was 9 patients who were never-smokers or former light-smokers (those who had stopped smoking at least 15 years previously and had a total of ≤ 10 pack-years of smoking), regardless of whether cancer tissue was available. The subsequent cohort was 8 patients who were also never-smokers or former light-smokers and, in addition, required the availability of cancer tissue for genetic analysis and IHC.

### Germline exome sequencing

The methodological details are described elsewhere^22^. In brief, DNA was isolated from peripheral blood mononuclear cells using the QIAamp DNA Blood Mini Kit (Qiagen) and then an Ion Torrent adaptor-ligated exome library was generated by following the manufacturer’s protocol (Ion AmpliSeq Exome RDY Kit PIv3, Rev. A.0; MAN0010084, Thermo Fisher Scientific). Sample emulsion PCR, emulsion breaking, and enrichment were performed using the Ion Chef (Thermo Fisher Scientific). Template-positive ISPs were enriched, and sequencing was performed using Ion PI Chip v3 chips on the Ion Torrent Proton. Data were initially processed using Ion Torrent platform-specific pipeline software, Torrent Suite v4.0 (Thermo Fisher Scientific), to generate sequence reads, trim adapter sequences, filter, and remove poor signal-profile reads. Initial variant calling from the Ion AmpliSeq sequencing data was generated using Torrent Suite. To eliminate erroneous base calling, two filtering steps were used to generate final variant calling. The first filter was set at an average depth of total coverage of > 50, with each variant coverage > 15 and P-value < 0.01. The second filter was employed by visually examining mutations using Integrative Genomics Viewer software (http://www.broadinstitute.org/igv) or CLC Genomics Workbench v12.0.0 (Qiagen).

To refine the target genes for further study, we identified genes in which a truncating mutation (nonsense mutation, splice-site mutation, or frameshift indel) was observed in at least two cases^23^. We excluded variants with allele frequencies greater than 0.5% in a control reference group (Genome Aggregation Database: gnomAD)^24^. The strategy failed to identify the candidate genes. As a way of an alternative option, we surveyed the germline variants for 55 cancer susceptibility genes (Table 1).

**Table 1:** Gene list used for analysis (n = 55).

*AKT1* (MIM: 164730)	*EGFR* (MIM: 131550)	*NF1* (MIM: 613113)	*SDHAF2* (MIM: 613019)
*APC* (MIM: 611731)	*ERBB2* (MIM: 164870)	*NF2* (MIM: 607379)	*SDHB* (MIM: 185470)
*ATM* (MIM: 607585)	*FAM175A* (MIM: 611143)	*PALB2* (MIM: 610355)	*SDHC* (MIM: 602413)
*ATR* (MIM: 601215)	*FH* (MIM: 136850)	*PARK2* (MIM: 602544)	*SDHD* (MIM: 602690)
*BAP1* (MIM: 603089)	*FLCN* (MIM: 607273)	*PIK3CA* (MIM: 171834)	*SMAD4* (MIM: 600993)
*BARD1* (MIM: 601593)	*KIT* (MIM: 164920)	*PMS2* (MIM: 600259)	*STK11* (MIM: 602216)
*BMPR1A* (MIM: 601299)	*KLLN* (MIM: 612105)	*POLE* (MIM: 174762)	*TERT* (MIM: 187270)
*BRCA1* (MIM: 113705)	*MEN1* (MIM: 613733)	*PTEN* (MIM: 601728)	*TP53* (MIM: 191170)
*BRCA2* (MIM: 600185)	*MLH1* (MIM: 120436)	*RAD51C* (MIM: 602774)	*TSC1* (MIM: 605284)
*BRIP1* (MIM: 605882)	*MRE11A* (MIM: 600814)	*RAD51D* (MIM: 602954)	*TSC2* (MIM: 191092)
*CDH1* (MIM: 192090)	*MSH2* (MIM: 609309)	*RB1* (MIM: 614041)	*VHL* (MIM: 608537)
*CDKN2A* (MIM: 600160)	*MSH6* (MIM: 600678)	*RECQL2* (MIM: 604611)	*WT1* (MIM: 607102)
*CHEK2* (MIM: 604373)	*MUTYH* (MIM: 604933)*	*RET* (MIM: 164761)	*YAP1* (MIM: 606608)
*DICER1* (MIM: 606241)	*NBN* (MIM: 602667)	*SDHA* (MIM: 600857)	

*Considered to be associated with tumor predisposition in the homozygous or compound-heterozygous state.

### Sanger sequencing

To confirm the variants identified by next-generation sequencing (NGS), polymerase chain reaction (PCR) amplification and Sanger sequencing were performed using standard reagents and conditions, which are described elsewhere^25^. The sequences of the primers used in these experiments are described in Supplemental Table S1.

### Target NGS and targeted variant sequencing of DNA from paraffin-embedded tissue

DNA was extracted from paraffin sections of tumour tissue using the Maxwell RSC Kit and DNA FFPE Kit (Promega) by following the manufacturer’s guidelines. Target NGS with primer sets of all exons and promoter regions for four genes, *MLH1*, *PMS2*, *MSH2*, and *MSH6*, was performed on formalin-fixed paraffin-embedded (FFPE) normal tissue DNA. We used the Ion Torrent Proton for NGS. Thereafter, the variants were identified in the same manner as in the case of the exome sequencing described above. The variants found by NGS were confirmed by the Sanger sequencing in both directions. The sequences of the primers used in these experiments are described in Supplemental Table S1.

### Microsatellite instability (MSI) analysis

MSI analyses were performed on genomic DNA extracted using the Maxwell RSC FFPE DNA Kit and Maxwell RSC Instrument (Promega). MSI analyses were performed using the MSI Analysis System v1.2 (Promega). The analysis included the 5 mononucleotide markers BAT-25, BAT-26, NR-21, NR-24, and MONO-27 (Promega, MSI Analysis System v1.2). The PCR products were separated by capillary electrophoresis (Thermo Fisher Scientific) and analysed by GeneMapper software v4.1 (Applied Biosystems).

### Immunohistochemistry

For IHC of MLH1, PMS2, MSH2 and MSH6, FFPE tissue sections were collected from the patients. All specimens were cut into 4-μm-thick slices. The slides were incubated with mouse monoclonal antibodies against MSH2 (ready to use; clone FE11; Dako/Agilent), MLH1 (ready to use; clone G168-15; Dako/Agilent) or PMS2 (ready to use; clone A16-4; Ventana/Roche). Regarding MSH6, rabbit monoclonal antibody (ready to use; clone SP93; Ventana/Roche) was used. Cases were recorded as positive for expression when nuclear staining was present in all tumour cells. Cases were scored as negative for expression when all tumour cells showed complete loss of staining, provided that the normal cells around the tumour showed nuclear staining. Heterogeneous staining was defined according to the criteria established by Joost et al*.* as tumours showing intraglandular heterogeneity (strongly immunoreactive cells admixed with negative cells) and/or zonal loss (confluent areas of staining loss involving multiple adjacent glands)^26^.

## Results

We conducted exome sequencing of DNA extracted from leukocytes. Of 9 sporadic patients with NSCLC and NMTC, two patients harboured *MSH6* missense mutations, none of which were present in the control reference gnomAD database at an allele frequency over 0.5%. An *MSH6* heterozygous variant (all positions refer to genome build hg19), chr2:g.48032109 C>T; NM_000179.2:c.3499 C>T, p.Leu1167Phe, was identified by NGS and verified by Sanger sequencing in a female patient in her 50 s (case A). This variant was absent from the gnomAD and was predicted to be deleterious using in silico analyses (Table 2). Another heterozygous variant, chr2:g.48027683 A>T; NM_000179.2:c.2561 A>T, p.Leu854Met, was identified in a female patient in her 40 s (case B). This variant was reported in the gnomAD database with an allele frequency of 3.74 × 10^–4^ (1.66 × 10^–3^ in East Asian subpopulation). This variant was also presumed to cause dysfunction using in silico analyses.

**Table 2:** In silico bioinformatics analyses of the identified variants.

Analysis	InSiGHT classification	SIFT	polyphen2	Mutation assessor	PON-MMR2	FATHMM inherited disease	GERP RS score
chr2: 48032109 C>T (MSH6: Leu1167Phe)	(Not classified)	0.055 (tolerated)	0.529 (possibly damaging)	2.08, medium	0.456 (neutral)	− 2.25, damaging	4.15
chr2: 48027683 A>T (MSH6: Lys854Met)	(Not classified)	0.002 (damaging)	1 (probably damaging)	3.14, medium	0.586 (pathogenic)	− 2.83, damaging	4.48
chr3: 37053562 C>T (MLH1: Arg217Cys)	InSiGHT:3	0.001 (damaging)	1 (probably damaging)	3.585, high	0.904 (pathogenic)	− 1.91, damaging	5.76
chr3: 37035011 A>G (MLH1: − 28 A>G)	InSiGHT:3	(na)	(na)	(na)	(na)	0.070386, benign (FATHMM-XF)	− 9.1199

FATHMM-XF: non-coding score included.

**Figure 1:**
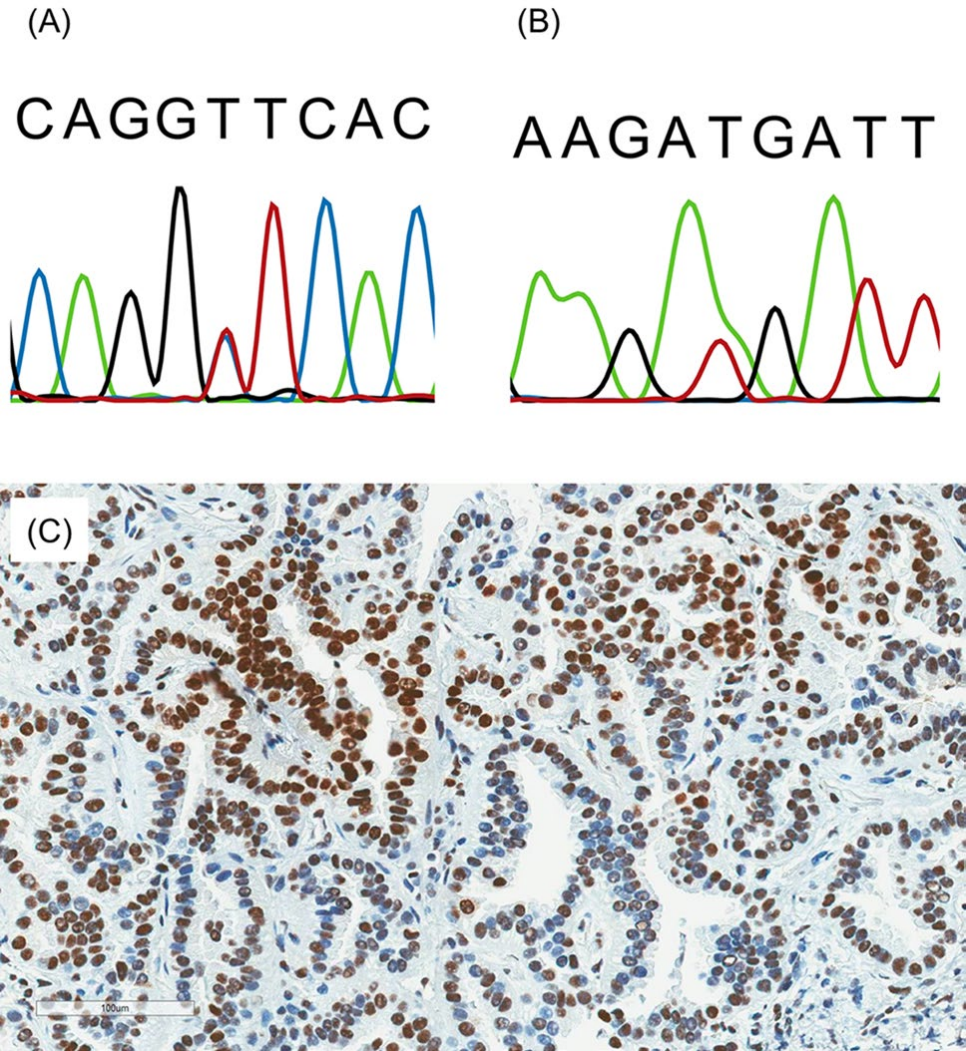
Sanger validation and MSH6 heterogeneous staining in case A and case B. (**A**) Sanger sequencing of the variant (chr2:g.48032109 C>T; NM_000179.2:c.3499 C>T, p.Leu1167Phe), in case A. (**B**) Sanger sequencing of the variant (chr2:g.48027683 A>T; NM_000179.2:c.2561 A>T, p.Leu854Met), in case B. (**C**) Case A showed heterogeneous staining of MSH6 with intraglandular loss (biopsy specimen of non-small-cell lung cancer).

NSCLC tissue of the case with the Leu1167Phe germline variant (case A) was available. Conventional IHC assays for MSH2 and MSH6 were performed and showed a heterogeneous staining pattern (intraglandular heterogeneity) in terms of MSH6 protein (Fig. 1). MSI was tested, but the tumour showed an MSI-stable phenotype. In both of these cases, the family history of malignancy did not meet the Bethesda criteria, and there was no previous history of colorectal or endometrial cancer, which is associated with Lynch syndrome.

We conducted further analysis of sporadic cases of NSCLC and NMTC. Histologically, all NSCLCs were lung adenocarcinoma, and all NMTCs were papillary thyroid carcinoma. A total of 8 cases were analysed in detail (Table 3). Tumour tissue originating from organs other than the lung was also analysed when available. A single nucleotide variant was identified in 2 out of 8 patients. A germline heterozygous *MLH1* gene variant (chr3:g.37053562 C>T; NM_000249.3: c.649 C>T, p.Arg271Cys) was identified from patient 1, who had a history of lung adenocarcinoma and papillary thyroid cancer. This variant was reported in the gnomAD database with an allele frequency of 3.4 × 10^–4^ (4.41 × 10^–3^ in East Asian subpopulation). Patient 7 had a germline heterozygous gene variant located in the *MLH1* promoter region (chr3:g.37035011 A >G; NM_000249.3:c. − 28 A> G), with an allele frequency of 1.67 × 10^–3^ in the gnomAD (4.81 × 10^–3^ in East Asian subpopulation).

**Table 3:** Demographic, genetic and histologic characteristics with MMR immunohistochemistry.

Case	Sex	Cancer type	Age at diagnosis	Family history	Germline variants	IHC status	Driver gene alterations
1	Female	Lung Ca.	60s	None	MLH1 Arg217Cys	Lung: no loss of staining	EGFR exon 21 L858R
		Thyroid Ca.	50s				
2	Female	Breast Ca.	60s	Concealed at the request of the patient	Not identified	Lung: no loss of staining	EGFR exon 21 L858R
		Lung Ca.	60s				
		Thyroid Ca.	60s				
3	Male	Lung Ca.	60s	(Unknown)	Not identified	Lung: heterogeneous staining in MSH2 and MSH6 (intraglandular pattern in both protein)	No major driver alterations*
		Thyroid Ca.	60s				
4	Male	Gastric Ca.	60s	(Unknown)	Not identified	Gastric: heterogeneous staining in PMS2 (intraglandular pattern) Lung: no loss of staining	KRAS exon 2 G12V
		Lung Ca.	60s				
		Thyroid Ca.	60s				
5	Female	Lung Ca.	60s	None	Not identified	Lung: no loss of staining Thyroid: heterogeneous staining in MSH6 (intraglandular pattern)	EGFR exon 19 deletion
		Thyroid Ca.	60s				
6	Male	Lung Ca.	60s	Sister: colon cancer	Not identified	Lung: no loss of staining	EGFR exon 21 L858R
		Prostate Ca.	60s				
		Thyroid Ca.	60s				
7	Female	Lung Ca.	50s	None	MLH1 -28 A>G	Lung: no loss of staining	EGFR exon 19 deletion
		Thyroid Ca.	50s				
8	Female	Lung Ca.	50s	Sister: gastric cancer	Not identified	Lung: heterogeneous staining in MLH1 (intraglandular) and PMS2 (intraglandular)	EGFR exon 20 insertion
		Thyroid Ca.	60 s				

*Negative results (NGS on validated platforms) for oncogenic driver alteration: EGFR (exon 18–21) activating mutation, HER2 (exon20) activating mutation, KRAS mutation, BRAF (exon 15) mutation, MET amplification, ALK rearrangement or ROS1 rearrangement.

**Figure 2:**
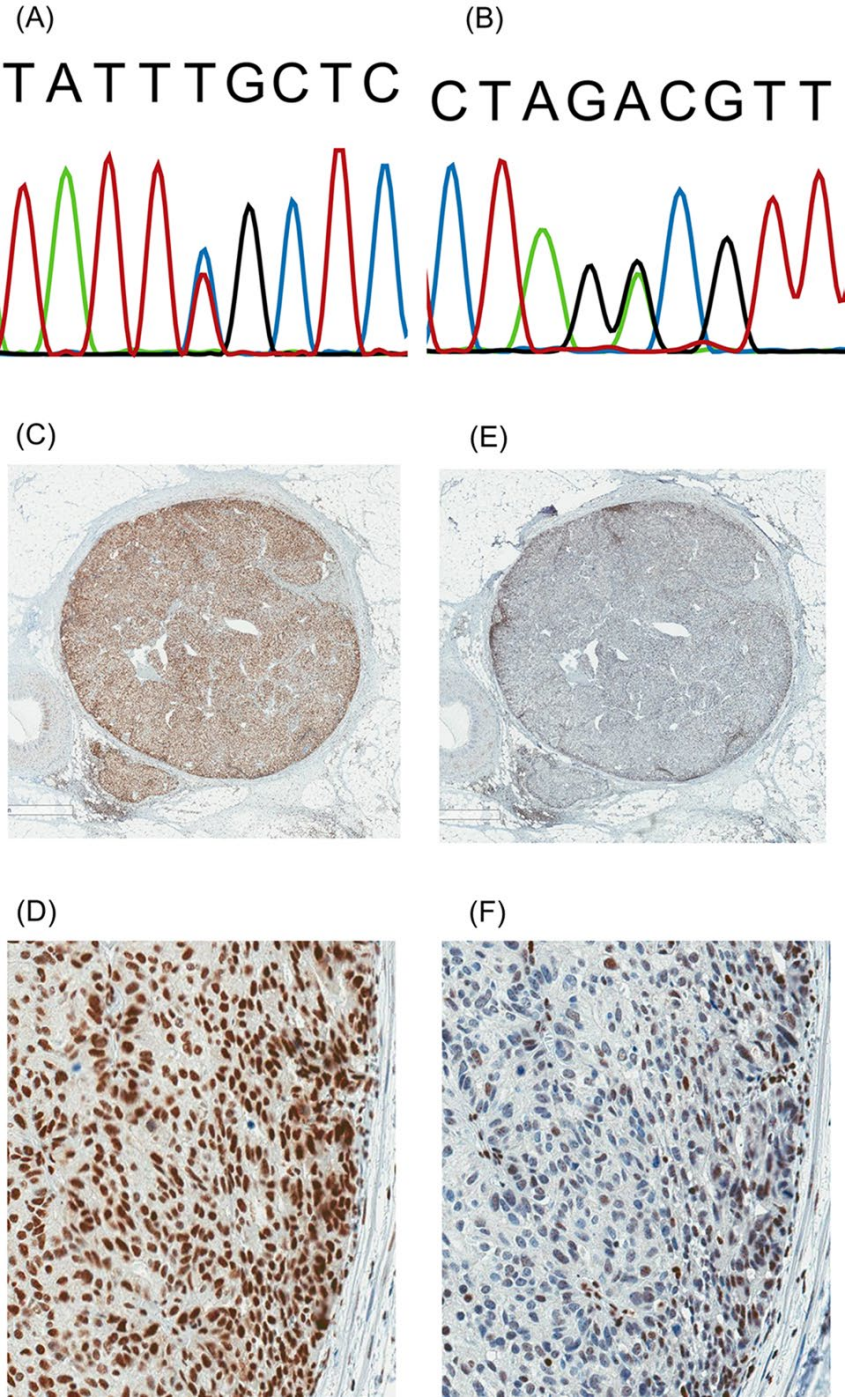
MMR gene variants and IHC heterogeneity in the second cohort. (**A**) Sanger sequencing of the variant (chr3:g.37053562 C>T; NM_000249.3: c.649 C>T, p.Arg271Cys), in case 1. (**B**) Sanger sequencing of the variant (chr3:g.37035011 A>G; NM_000249.3:c. − 28 A>G), in case 7. (**C**–**F**) Case 4 showed intact MLH1 (**C**,**D**), with heterogeneous staining of PMS2 (intra-glandular loss in (**E**,**F**)) in a gastric cancer specimen.

Ten primary tumour specimens were obtained and were evaluable from 8 patients. Four out of ten specimens showed heterogeneous MMR protein expression. As shown in Table 3, the heterogeneous pattern was different in each tumour. All cases of “staining heterogeneity” showed an intraglandular pattern (Fig. 2). Regarding MSI, all of the obtained cancer specimens were microsatellite stable.

## Discussion

A germline mismatch repair gene variant determined to have impaired function was detected at a high frequency in our cohort. An uncommon, functionally deficit variant was observed in 2 out of 9 patients who underwent germline exome sequencing and 2 out of the 8 cases in subsequent exploration phases. However, none of these mutations have been proven to cause Lynch syndrome. This fact is in line with our observation that no patients showed clinically typical Lynch features in the study; no patients had a history of colon cancer or endometrial cancer. Moreover, Lynch syndrome was not strongly suspected from the family histories of the patients.

A heterogeneous pattern in MMR immunostaining in cancer specimens was associated with reduced MMR capacity. Watson et al*.* conducted a population-wide study that used MSI as the pre-screen test followed by IHC for positive cases^27^. They screened for MSI in all colorectal cancers diagnosed in Western Australia throughout a 5-year period in patients < 60 years of age. MSI was found and Lynch syndrome suspected in 69 cases. All cases underwent IHC analyses and seven cases were classified as having abnormal staining because of heterogeneous patterns of MMR loss. Three of these seven cases had previously been found to have germline mutations. Joost et al*.* reported heterogenous staining patterns that affected at least one of the mismatch repair proteins MLH1, PMS2, MSH2, and MSH6, which were identified in 14 colorectal cancers^26^. Differences in MSI or *MLH1* methylation patterns were observed in these tumours. Recently, several investigators have separately isolated IHC-retained tumours and IHC-lost tumours, extracted the DNA, and performed NGS and MSI analyses. They reported different molecular profiles and/or MSI statuses in a substantial number of cases^28^. In our study, 6 out of 9 patients who had a tissue specimen, including exome-sequenced cases, showed a tumour with a heterogeneous staining pattern, which implies suboptimal mismatch repair status in the tumour.

Although none of the samples were found to be MSI-high, it cannot be concluded that MMR gene dysfunction was not related to patients included in our study. Even when analysing tumour samples from patients with established Lynch syndrome (namely, a germline pathogenic variant had already been identified), there is a difference in the frequency of MSI-high between colorectal cancer and endometrial carcinoma^29^. Moreover, MSIs for tumours other than these two are more likely to be stable^30^. There have been several reports of cases in which a germline pathogenic variant was identified, in which a tumour other than Lynch spectrum had developed and the MSI was stable, despite loss of MMR protein by immunohistochemistry expression. In the past, when MSI-stable tumours were identified in patients with Lynch syndrome, it was usually concluded that these tumours were coincidental and not related to germline MMR protein defects. However, several researchers have suggested that tissue-specific factors other than germline MMR gene defects are required to produce MSI because MSI-stable endometrial carcinoma is seen in a considerable number of patients with Lynch syndrome. Although the rates of MSI-high in NSCLC and thyroid cancer are approximately 1% and 0–2%^31,32^, respectively and our result is consistent with the literature, MMR dysfunction may have been involved in the development of neoplasms in our cases.

Human lung cancer pathophysiology and suboptimal DNA repair capacity (DRC) have been linked in the literature. Wei et al*.* hypothesized that humans differ in their ability to repair DNA against tobacco-causing carcinogens and other substances that cause DNA damage, which may be related to the development of lung cancer, and they conducted a case–control study of 316 newly diagnosed lung cancer patients and 316 cancer-free control subjects^33^. DRC was measured in cultured lymphocytes with the use of the host-cell reactivation assay with a reporter gene damaged by a known tobacco carcinogen, benzo[α]pyrene diol epoxide. They showed that lower DRC was observed more frequently in case patients than in control subjects (P < 0.001) and was associated with a greater than twofold increased risk of lung cancer^33^. A similar study, limited to lung cancer in lifetime never-smokers found a 3.38-fold risk for individuals with a DRC below the first quartile (95% CI 1.8–6.3) compared with individuals with a DRC above the third quartile^34^. In the present study, cases with a variant of the DNA repair gene and a heterogeneous pattern of immunohistochemically stained MMR in cancer specimens were likely to be in a suboptimal DRC state, which is presumed to have caused lung cancer.

Traditionally thyroid cancer is not considered to be part of the Lynch syndrome tumour spectrum; however, several reports have indicated an association between MMR insufficiency and thyroid cancer. Stulp et al*.* reported a 44-year-old woman with Lynch syndrome who developed thyroid cancer^35^. Genetic analysis of the *MSH2* gene in the patient revealed the c.1704_1705delAG mutation. The thyroid cancer tissue showed complete loss of immunohistochemical expression of the MSH2 and MSH6 proteins in the presence of normal positive internal controls and no loss of the MLH1 and PMS2 proteins. Yu et al*.* evaluated the underlying genetic causality of familial NMTC, defined by the diagnosis of two or more first-degree relatives affected by differentiated thyroid cancer of follicular cell origin. Using NGS with a customized panel to capture 31 cancer susceptibility genes, they performed deep sequencing of 47 familial patients, and several germline mutations were found to match between paired familial NMTC patients from the same family, including *MSH6* G355S and A36V and *MSH2* L719F^36^.

From an embryological point of view, the human thyroid and lungs originate as neighbouring bud-shaped outgrowths from the midline of the anterior embryonic foregut during normal organogenesis. Studies with murine models and pluripotent stem cells have indicated that some aspects of the development of these organs involve similar gene sets^37–40^.

It is not clear whether the previous cases described here represented a subtype of Lynch syndrome or were presumed to be based on insufficient MMR capacity but a different group of diseases other than Lynch syndrome. Looking back on family G from the 1913 study, gastric cancer was the third most common (8 persons) in the descendants of the family^41^. This is said to be due to the lack of water supply and refrigerators at that time, and other risk factors such as *H. pylori* and cured meat contributing to the relatively high incidence of gastric cancer. In the future, as the prevalence of various cancers in the general population fluctuates, the incidence of Lynch syndrome spectrum cancers may change. Increasing trends in never-smoker lung adenocarcinoma in developed countries may alter the future Lynch syndrome cancer spectrum. In the landmark report from Dr. Lynch in 1971, two family members developed lung cancer, and one of them had multiple primary malignancies: lung cancer and thyroid cancer. These cancers might be observed more frequently in the future. We also speculate that the risk of receiving a diagnosis of different types of cancer varies throughout a person’s life span. Most of the patients in our study developed lung adenocarcinoma and/or thyroid carcinoma in their 60 s. Generally, the average age of cancer onset in Lynch syndrome is approximately age 50, which is decades earlier than the average age seen in our cohort^42^. It is unclear what this difference means.

This study had several limitations. First, this was a single-institution retrospective study conducted in Japan, and there were considerable geographical effects. Second, the coexistence of NSCLC and NMTC, either synchronously or metachronously, is relatively rare, and we had to perform our analysis with a limited number of cases.

The results of our analysis implied causal roles of impaired MMR capacity in NSCLC and NMTC. Continuing investigations in patients with NSCLC and NMTC, especially never-smokers, young patients, or patients with a family history, will clarify the pathophysiology of this condition. Additionally, the results described here may be useful for elucidating the pathology of smoking-unrelated lung adenocarcinoma, which has become a cause of substantial global burden in recent years.

## Supplementary Information

Supplementary Information.

## Abbreviations

95% CI: 95% Confidence interval
ALK: Anaplastic lymphoma kinase
DRC: DNA repair capacity
EGFR: Epidermal growth factor receptor
FFPE: Formalin-fixed paraffin-embedded
gnomAD: Genome aggregation database
IHC: Immunohistochemistry
MMR: Mismatch repair
MSI: Microsatellite instability
NGS: Next-generation sequencing
NMTC: Nonmedullary thyroid cancer
NSCLC: Non-small-cell lung cancer
