# Supplementary material for: Alteration of DNA mismatch repair capacity underlying the co-occurrence of non-small-cell lung cancer and nonmedullary thyroid cancer
Source: Sci Rep. 2021 Feb 11;11:3597. doi: 10.1038/s41598-021-83177-1 (PMC7878500; doi:10.1038/s41598-021-83177-1)
Supplement: Supplementary file 1 — Supplementary Information. [file 41598_2021_83177_MOESM1_ESM.docx]

Supplementary information

**Alteration of DNA mismatch repair capacity underlying the co-occurrence of non-small-cell lung cancer and nonmedullary thyroid cancer**

Shiro Fujita MD, PhD^a,b^, Katsuhiro Masago MD, PhD^b,c^

a. Department of Respiratory Medicine, Kobe Central Hospital, 2-1-1 Soyama-cho, Kita-ku, Kobe, Hyogo, 651-1145, Japan

b. Division of Integrated Oncology, Institute of Biomedical Research and Innovation, 2-2 Minatojima Minami-machi, Chuo-ku, Kobe, Hyogo, 650-0047, Japan

c. Department of Pathology and Molecular Diagnostics, Aichi Cancer Center, 1-1 Kanokoden, Chikusa-ku, Nagoya, Aichi, 464-8681, Japan

Corresponding author: Shiro Fujita MD, PhD

E-mail: jp.shirofujita@gmail.com

Supplementary Table 1. Summary information of the conventional PCR primer sets and their product sizes

| Variant | Forward primer | Reverse primer | Product size (bp) |
| --- | --- | --- | --- |
| chr2: 48032109 C>T  (MSH6: Leu1167Phe) | AAGACCTTTTCCTCCCTCATTCA | TGACTGAATGAGAACTTAAGTGGG | 183 |
| chr2: 48027683 A>T  (MSH6: Lys854Met) | GAAGAGTCAGAACCACCCAGA | AAAACCATCAGCAACTTCTTCCA | 151 |
| chr3: 37053562 C>T  (MLH1: Arg217Cys) | CTTGTGTCTTCTGCTGTTTGTTTA | AGCCTGTGTATTTGACTAAAGCAA | 246 |
| chr3: 37035011 A>G  (MLH1: -28 A>G) | CTACAGCTGAAGGAAGAACGTG | TCCGTACCAGTTCTCAATCATCT | 224 |
